# Novel Model of Childhood Appetitive Traits in Children with Obesity

**DOI:** 10.21203/rs.3.rs-5318259/v1

**Published:** 2024-11-15

**Authors:** Vidhu Thaker, Sunaina Nigam, Mengqi Zhu, Ashley Shoemaker, Amy Gross, Claudia Fox

**Affiliations:** Columbia University Irving Medical Center; Columbia University; Mailman School of Public Health; ashley.h.shoemaker@vumc.org; University of Minnesota Medical School; University of Minnesota

## Abstract

**Background/Objectives:**

Appetitive traits have a central role in energy intake and development of obesity. Child Eating Behavior Questionnaire (CEBQ) is a validated psychometric tool to measure appetitive traits in children. This study sought to assess the CEBQ factor structure in children with obesity. We hypothesized that hedonic traits are more prominent with obesity and in older children.

**Subjects/Methods:**

This cross-sectional observational study used CEBQ data from three geographically diverse centers in children with obesity. Eight known CEBQ subscales and the established factor model were compared by severity of obesity, age and sex. Exploratory factor analysis (EFA) to define the appetitive trait factor structure was undertaken in a training dataset and validated in a test set.

**Results:**

Children with obesity (n=814) showed higher food response, enjoyment of food, emotional overeating and desire to drink compared to population-based controls (p < 0.001) that correlated with obesity severity. The EFA identified a novel six-factor model with a new “Food avidity” factor that explained 15.8% of the variance. Satiety responsiveness (p < 0.001) and emotional undereating (p = 0.03) were lower in youth over six years compared to younger, while food fussiness and food avidity were the same, without any sex differences.

**Conclusions:**

CEBQ is useful to assess appetitive traits in children with obesity. The novel factor structure in youth with obesity suggests that temporal or phenotypic differences necessitate a revision of the established factor model. The new factor structure may be used to develop a hyperphagia questionnaire.

## Introduction

In 2020, 19.7% of the youth aged 2–19 years (14.7 million) had obesity (BMI ≥ 95th percentile)^1^ and 6.1% (4.5 million) had severe obesity (BMI ≥ 120% of 95th percentile)^2^ placing them at higher risk for long-term cardiometabolic disease and comorbidities. This rising prevalence is linked to biological, psychological, and social/environmental contributors. The behavioral susceptibility theory postulates that the genetic risk of obesity operates through appetitive traits, such that the individuals who are predisposed to obesity will have magnified responses to food environment, be more responsive to food cues and likely to continue to eat longer^3^. Defining the appetitive behavior patterns in response to accessible, calorically dense foods, may provide tools for intervention at individual and population levels.

Appetitive traits among children with and without obesity are shown to be related to caloric intake. As an example, high satiety responsiveness, slow eating, and food fussiness were found to be negatively associated with weight^4, 5^. Conversely, increased adiposity has been positively correlated to increased food-cue responsiveness, enjoyment of food, emotional overeating, and desire to drink^6, 7^. Further, children who eat more processed snacks, an appetitive trait related to these behaviors, have a higher risk of obesity^8, 9^. While these behaviors broadly distinguish between children with and without obesity, more research is needed on the specific behaviors associated with severe obesity.

The Children’s Eating Behavior Questionnaire (CEBQ) is a widely utilized 35-question, parent-completed psychometric tool to measure eating behavior in children from infancy to late childhood^10^. The original subscales and factor model was developed and validated in a community-based study in children from middle class without obesity in the United Kingdom^10, 11^. We and others have shown that children with obesity have measurable differences in the CEBQ subscales by increasing severity of obesity^6, 12^. While the CEBQ has been widely utilized across populations, it is not yet known if the existing factor structure applies to children with obesity and severe obesity in diverse populations. In this study, we sought to assess the CEBQ factor structure in children with varying obesity severity. Based on existing literature, we hypothesized that hedonic behaviors, as captured by food approach subscales, will be prominent in our cohort of children with obesity, and that younger children will have higher CEBQ derived homeostatic response to food cues, assessed by food-avoidance related subscales.

## Methods

This cross-sectional study used data collected from participants from pediatric weight management programs from three geographically diverse US tertiary care medical centers, either for clinical care or in research studies, between 2010 and 2017. The parent/caretaker of each patient/participant completed the questionnaires related to clinical care or research with the appropriate consent. Inclusion criteria were ages 2–12 years, presence of obesity (BMI ≥ 95th percentile) at the time of enrollment, and available CEBQ data. The data use was approved by the Institutional Review Board (IRB) at each respective institution. The data were aggregated under appropriate data use agreements for analysis and approved by the IRB at Columbia University (AAAR0351).

### Study populations

Boston Children’s Hospital (BCH): a) Genetics of early childhood (GECO) study is an observational study to investigate the genetic underpinnings of severe early onset (prior to 6 years of age) in children and adolescents (Clinicaltrials.gov, NCT01998750). The child and caregiver(s) attended a clinical visit at the research unit where demographic information, physical measurements such as height, weight, blood pressure and physical examination were performed. CEBQ was completed by the caregiver accompanying the child to the visit for children < 13 years of age. b) POOL Research Obesity Registry (named after the four clinical recruitment sites) is a longitudinal observational study of children with BMI ≥ 85th percentile^13^. Demographics, anthropometrics, CEBQ and measures of energy expenditure, and body fat were obtained at a research visit at the enrollment visit for children < 13 years of age.

Vanderbilt Medical Center (VMC): This longitudinal observational registry enrolled children with obesity at a weight management program. Demographic details, physical measurements, and behavioral questionnaires including CEBQ were obtained at the time of enrollment.

University of Minnesota (UMN): The Pediatric Weight Management clinic is a multidisciplinary clinic with four locations. CEBQ was obtained from patients at their initial clinical visit along with other demographic and clinical measurements.

### Measures

The Children’s Eating Behavior Questionnaire (CEBQ) is a validated 35-item questionnaire that measures eating behaviors in children on a five-point Likert scale (1 = never, 5 = always) completed by a parent/caregiver^10^. In the initial CEBQ validation study, the 35 items were split into eight subscales: “Food Responsiveness”, “Enjoyment of Food”, “Emotional overeating”, “Desire to Drink”, “Satiety Responsiveness”, “Slowness in Eating”, “Emotional Under-eating”, and “ Food Fussiness”^11^. Four food responsive subscales – food responsiveness, enjoyment of food, emotional overeating and desire to drink– comprise of the food approach or “Hedonic” subscales, while the other four – satiety responsiveness, slowness in eating, emotional undereating and food fussiness, are suitable as food avoidant or “homeostatic” subscales. These subscales were collapsed into a seven-factor model where food responsiveness, enjoyment of food, emotional overeating, desire to drink, emotional undereating, and food fussiness mapped to their own factor while slowness in eating and satiety responsiveness loaded on to a single factor^11^. The eight subscales and the seven-factor model of the CEBQ described by Wardle^10^ and Carnell et al^11^ have been extensively used around the world in youth with and without obesity^12^.

### Additional data

Demographic and anthropometric data were obtained either from the research study or from the Electronic Health Records at the respective institution. Self-reported race and ethnicity were obtained and combined into one category. A report of Hispanic/Latinx ethnicity was prioritized into Hispanic/Latino category, whereas others were grouped based on self-identified race into Non-Hispanic White (NHW), Non-Hispanic Black (NHW), and others (Other). The severity of obesity was defined based on the CDC revised 2022 criteria for classification of obesity – Class 1 includes BMI between 95th and 120% of the 95th percentile, Class 2 between 120% − 140% of the 95th percentile and Class 3 ≥ 140% of the 95th percentile^14^. Class 2 and 3 are considered severe obesity.

### Data Analysis

CEBQ was scored based on the guidance from its development papers^10, 11^. Briefly, for the comparison of the subscales, five questions are reverse scored, e.g. Q.3 “My child has a big appetite” in SR, and Q #10 “My child enjoys tasting new foods” in FF. Multiple imputation was done using multivariate imputation by chained equation using mice package in R statistical language^15^ for 238 (3.6%) of the missing CEBQ subscales. Independent t-tests were utilized to compare means and standard deviations of the eight CEBQ subscales in our study cohort with obesity compared to a simulated sample based on the correlation matrix from the original validation sample by Wardle et al^10^. Differences in the appetitive traits by sex, and age (≤ 6 years, > 6 years) were evaluated by independent sample t-tests and MANOVAs. The trend of the appetitive behaviors by obesity class was assessed by Mann-Kendall trend test. Sensitivity analysis was done by including the age and sex in linear regression models. Based on the prior literature, we performed confirmatory factor analyses (CFA) with the 7-factors (original Wardle et al model) and 8-factors (CEBQ subscales)^10^. Model fit was assessed using the root mean square error of approximation (RMSEA), the comparative fit index (CFI), the Tucker-Lewis Index (TLI), and standardized root mean square residual (SRMR). Modification indices were examined. The *a priori* model fit criteria were: RMSEA < 0.5 for good fit and < 0.8 for acceptable fit, CFI/TLI > 0.9, and SRMR < 1.0^16, 17^. Subsequently, an exploratory factor analysis (EFA) to assess the best-fit model for the data from our cohort was performed. The cohort was randomly split into a training (2/3) and a testing set (1/3) including participants from each cohort. Parallel analysis scree plot was used to identify the components and factors for EFA. Factor loadings of > 0.3, parsimony, and factor structure were used to define the factors. The factor structure identified in the training dataset was validated in the testing set by CFA using the same fit criteria as above with correlation structure based on modification indices. The statistical analyses were done in R-statistical software v 4.3.2 and MPlus v 8.4. A p-value < 0.05 was considered statistically significant.

## Results

### Cohort features

**Figure 1:**
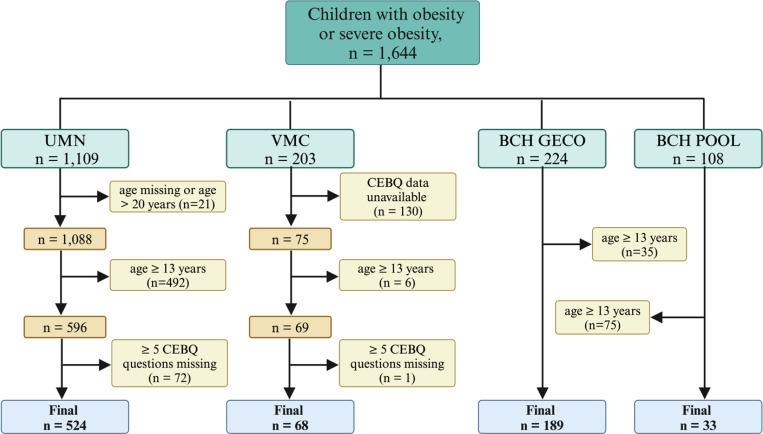
Flow of study participants for four cohorts from three institutions. Abbreviations: CEBQ, child eating behavior questionnaire; BMI, body mass index; BCH, Boston Children’s Hospital; VMC, Vanderbilt Medical Center; UMN, University of Minnesota; GECO, Genetics of Early Childhood Obesity study; POOL, Pediatric overweight and obesity registry named for the various participating clinics.

A total of 1 644 participants were available for the study, of which 814 were included in the analysis. The detailed participant flow for each institution is listed in Figure 1. The combined cohort and the demographic detail by institution is provided in Table 1. Overall, there was a higher proportion of individuals with severe obesity compared to Class 1 obesity (Class 1: 177 [22%], Class 2: 281 [35%], Class 3: 356 [44%], p < 0.001). By center, the sex distribution of participants was similar between BCH and UMN, but there were higher number of girls at VMC. There was a higher proportion of individuals of Hispanic ethnicity at BCH, who were also younger, and with more severe obesity. The CEBQ subscales of food responsiveness and food fussiness were similar across the three centers, but all other subscales were different (Table 1).

### Comparison of appetitive trait subscales with the historical control and between subgroups

**Figure 2:**
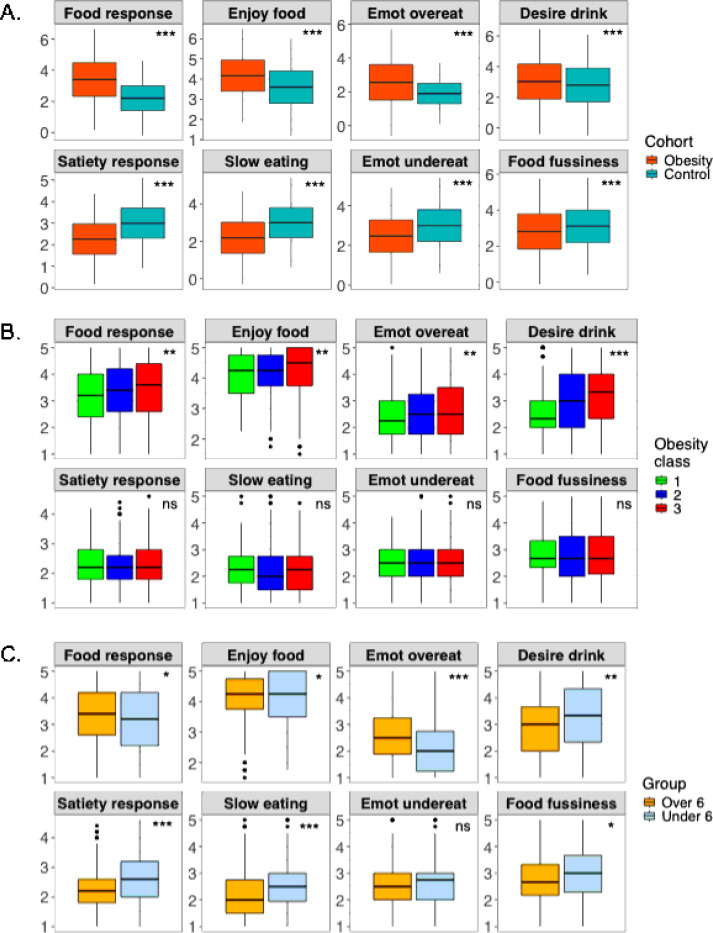
CEBQ subscales. A. comparison of the cohort with obesity and the original CEBQ subscales (Wardle 2001); B. by obesity class in the cohort with obesity; C. by age group over 6 years compared to the younger children. * p =0.05–0.01, ** p = 0.01–0.001, *** p < 0.001, ns = not significant. Abbreviations: Emot, emotional

There was good internal consistency in the CEBQ data from the obesity cohort (Cronbach’s alpha = 0.88). The CEBQ scores of the obesity cohort were higher on the hedonic subscales (food responsiveness, enjoyment of food, emotional overeating, desire to drink, p < 0.001) and lower for the homeostatic subscales (satiety responsiveness, slowness in eating, emotional undereating, food fussiness, p< 0.001) compared to the historical control from the original Wardle et al cohort (Figure 2 A). There was a positive trend for the hedonic subscales with increasing class of obesity (food responsiveness, p = 0.002; enjoyment of food, p=0.004; emotional overeating, p=0.004; desire to drink, p < 0.001). However, the homeostatic subscales did not show such a trend (p > 0.1, Figure 2 B). On comparing the CEBQ subscales for children over 6 years (n= 675) with those under 6 years of age (n= 139), the hedonic subscales were higher (food responsiveness, p = 0.02; enjoyment of food, p= 0.04; emotional overeating, p < 0.001; desire to drink, p = 0.002). The homeostatic subscales for children over 6 years were lower (satiety responsiveness, p < 0.001; slowness in eating, p < 0.001, food fussiness, p = 0.01) except emotional undereating subscale (p > 0.05) (Figure 2 C). On comparing by the sex, amongst the hedonic scales, desire to drink was marginally higher in boys (p=0.05). But in the homeostatic subscales, satiety responsiveness and slowness in eating were lower (p= 0.03, and p < 0.001 respectively) and food fussiness was higher (p=0.02) in boys (Figure S1). The trend noted by obesity class remained while adjusting for the age and sex. The CFA for the CEBQ seven-factor as well as eight-factor model from Wardle and colleagues was a poor fit for the obesity cohort based on the CFA goodness-of-fit indices: 7-factor model: c^2^ (degrees of freedom = 539) = 4491.411, p < 0.001, RMSEA = 0.096, CFI = 0.884, TLI = 0.872, and SRMR = 2.715; 8-factor model: c^2^ (degrees of freedom = 532) = 4724.227, p < 0.001, RMSEA = 0.094, CFI = 0.891, TLI = 0.878, SRMR = 2.605, indicating an unsuitable fit.

### EFA in the training set and validation in the test set

The obesity cohort was split into two datasets for the EFA – training (70%) and test (30%)– with participants from each institution in each set. The demographic details of the training (n=569) and the test set (n = 245) are presented in Table S1 in the Appendix. Parallel analysis suggested six components and seven factors for the data (Figure S2). Six, seven and eight factor EFA models were considered with final selection of the six-factor model based on the factor structure, fit indices, and parsimony. The 6-factor model explained 50.8% of the variance in the data. Factor 1 explaining the highest variance included questions primarily related to hedonic traits of food craving and food approach (Table 2). This novel factor that we labeled “Food avidity” (FA) is comprised of 10 questions that includes the questions covered by Enjoyment of food and Food responsiveness subscales with two additional related questions and explained 15.7% of the variance. Food fussiness (9.8% variance) and desire to drink (6.7% variance) factors remained the same as those in the Wardle model. Emotional overeating (7.3% variance) included the questions from the Wardle model with the addition of overeating when happy. Satiety responsiveness (6.0% variance) comprised of the questions included those from satiety responsiveness and slowness in eating subscales save for two questions thematically linked to food avidity. Lastly, emotional undereating (5.3% variance) included three questions that were included in the original model. The hedonic factors from this model showed a trend by class of obesity (food avidity, p < 0.001; emotional overeating, p =0.01; desire to drink, p < 0.001), but not for the homeostatic factors (Figure S3). These trends were maintained while adjusting for the age and sex. In the testing set, the CFA with modification indices suggested good fit of the model (c^2^ (degrees of freedom = 534) = 902.54, p < 0.001, CFI = 0.967, TLI = 0.963, RMSEA = 0.053 [95% CI 0.047, 0.059], p ≤ .05 = 0.20, SRMR = 1.058).

## Discussion

This study of a large cohort of children with obesity confirmed that the CEBQ is a useful instrument for measuring appetitive traits in youth with obesity and severe obesity. We found that the cohort with obesity had higher hedonic (food approach) CEBQ subscales and lower homeostatic (food avoidance) subscales when compared to children with normal weight. Within the classes of obesity, the hedonic subscales were positively correlated with increasing obesity, while the homeostatic subscales did not differ across the obesity classes. The data did not fit well to the known 7-factor CEBQ factor model. In EFA, we identified a new 6-factor model with a prominent hedonic “Food Avidity” factor that showed an increasing trend with severity of obesity, and with older age, but was not different by sex. We validated a good fit of the new factor model in an independent test set.

In our pilot study, we have shown that children with severe obesity have higher scores on hedonic and lower on homeostatic subscales when compared to those with obesity^6^. In the larger cohort of the present study, the linear differences across the class of obesity for the hedonic scores were significant, even when adjusting for the other measured contributors such as age and sex, but such differences were not observed in the satiety subscales. The CEBQ measured appetitive traits have been hypothesized to contribute towards obesity in youth^18^. Population-based twin studies have shown a strong concordance of the homeostatic traits in monozygotic twins both in the infancy^19^ as well as age early childhood^20^. Subsequent studies have also shown an association of the homeostatic traits with weight-related genetic variants^7^. A systematic review of 67 cross-sectional studies with complete or partial CEBQ subscales in children with and without obesity showed a positive association of the hedonic subscales and a negative association of homeostatic subscales with obesity^12^. The authors concluded that appetitive traits are the key behavioral mechanisms leading to excess weight gain. In addition, they hypothesized that adiposity by itself may be associated with changes in appetite over time, such that children of higher adiposity develop increasingly avid appetite. Our study focused on children with obesity provides evidence to support this postulate that for children with obesity, especially those with severe obesity, the food avidity factor is most prominent. This contrasts with the original model established in a community-based population without obesity where the most prominent factor explaining 28% of the variance included homeostatic subscales^10^. In our cohort of children with significant obesity, it is problematic to note that the homeostatic behaviors may have been overridden, such that there were no differences in these traits across the severity of obesity compared to those previously noted in studies of children with less severe obesity^12^. This observation aligns with the report by Stanley Schachter in 1968, that demonstrated that adults with obesity were more responsive to food cues and less sensitive to satiety^21^.

Based on the differences in the appetitive subscales as well as population differences of our study cohort with obesity compared to the children where the CEBQ was first derived, it was unsurprising that the factor models did not align. Similar observations have been noted in a prior study of demographically different, Growing Up in Singapore Toward healthy Outcomes (GUSTO) cohort^22^. Like the GUSTO cohort, a six-factor model fit our data better, and in the EFA, our food avidity scale collapsed two original CEBQ food approach subscales - enjoyment of food and food responsiveness – and included two questions from the homeostatic subscales. As noted in our study, the GUSTO study found no differences in the factors for desire to drink and food fussiness. It was also remarkable to note that food fussiness remained as prominent in children with obesity compared to those without, although it would be expected that positive energy balance leading to obesity may come with downregulation of this trait. Unlike our study cohort, the population-based GUSTO study did not have enrichment of obesity (BMI z-score between 0.06–0.08, 16% overweight/obesity). These concordant observations in demographically and phenotypically diverse cohorts may suggest a temporal evolution of the appetitive traits in youth in the 25 years since CEBQ was first reported.

This study noted significant differences in the appetitive traits between children younger and older than six years. The ^10^ subscales were higher, while the satiety-related subscales were lower in the older children. These findings suggest that even in children with obesity, there is greater homeostatic control at the younger age that is possibly overwhelmed by hedonic traits as the children grow and presumably have greater environmental influence. This observation suggests that interventions such as responsive feeding^23^ and attention to food cues earlier in life may hold the potential to preserve the homeostatic responses, likely protective against obesity. Longitudinal studies from early infancy of children with and without obesity are needed to confirm this proposed evolution of appetitive traits. This observation also supports the notion that younger children (for example under six years of age) with higher food avidity (when higher homeostatic control is expected) may potentially have underlying monogenic causes. Thus, genetic testing may be more useful in younger children with hyperphagia and early onset severe obesity, as recommended by the Endocrine Society guidelines for management of obesity in children and adolescents^24^.

One of the important findings of this study is the prominence of the hedonic factors and the persistent differences by increasing severity of obesity while adjusting for other observed confounders. We propose that the hedonic factors noted in this study (food avidity, emotional overeating, and desire to drink) may serve as an effective subset to provide the much-needed tool for evaluation of hyperphagia in childhood. The need for such a tool is of outgrowing importance now with the increasing availability of genetic testing for obesity. The factor structure noted in the training dataset was replicated in the validation testing cohort. However, validation of this novel factor structure is needed in independent studies. We hope that the findings of this study will serve as a foundation for future studies to validate a hyperphagia measure that can be used to screen for the presence of genetic (either monogenic or polygenic) causes of obesity.

The strength of this study is the large diverse cohort of children with obesity that allowed the validation of the novel model. The cohort was an adequate sample size to perform the CFA, EFA, and model fit assessments, because participant data was not used for more than one analysis. Additionally, the cohort demonstrated adequate BMI, ethnicity, and age variability. This study was limited by using a historical control group. Wardle et al. cohort data were obtained prior to 2001 from primary schools in the United Kingdom. This population is not only temporally, but also demographically different. However, the alignment of at least some of the factors suggests the validity of the questionnaire in our cohort, as has also been noted in other studies from different continents. Further, the alignment of our novel EFA model with the population-based GUSTO cohort validates the findings in the current times. The cross-sectional study design at a time point when children already have obesity confounds the interpretation on whether the appetitive traits are reflective of the biological tendency towards obesity or is reflective of the overridden satiety traits. Future longitudinal population-based studies, especially birth cohorts, specifically focused on children who develop obesity over time will be needed to understand the evolution of appetitive traits during the development and establishment of obesity. This study did not have access to data on social determinants of health or food access that hold the potential to influence eating behaviors and shaping appetitive traits. Finally, our convenience cohort was ascertained from hospital-based clinics for treatment seeking youth. Hence, the findings can certainly be utilized by the large population seen at medical facilities, but the generalizability of the findings to the population remains unclear. However, due to the diverse geographical and racial/ethnic distribution of the participants, it is likely to be applicable to large treatment seeking population.

The findings of this study have important implications for clinical care. Management of appetitive traits remains a key intervention target for both prevention and management of obesity. Promotion of responsive feeding early in life at primary care level^23^ or as eHealth intervention^25^ at public health level that help in shaping early life appetitive traits are an important strategy for prevention of obesity. Regulation of food cues based on appetitive traits have been shown to be an effective obesity management intervention in adults^26, 27^. A pilot study of 44 children between 8–12 years showed improvement in food responsiveness with interventions targeted towards food cues^28^. Longitudinal studies with behavioral intervention have shown greater weight loss maintenance in those with higher satiety responsiveness, compared to those with higher food responsiveness^29^. Ongoing studies focused on behavioral and emotional focused therapy that also includes families for adolescents^30^ and younger children^31^ may provide further evidence on the effectiveness of such interventions on altering BMI and appetitive traits in individuals with obesity. Finally, the newer obesity medications that hold the potential to alter food avidity and satiety responsiveness may serve as additional tools to assist in management of obesity in youth.

## Conclusions

In this large study of children with obesity, we have identified that the CEBQ is a useful tool for measuring appetitive traits in treatment seeking clinical cohort of youth with obesity or severe obesity across three different institutions in the United States. The cohort had higher hedonic and lower homeostatic traits compared to children with normal weight. While the hedonic traits continue to follow the linear increase with severity of obesity, the homeostatic traits were not different. A new six-factor model with prominence of hedonic traits was identified that may be used towards treatment strategies and/or developing novel tools for management of obesity.

## Supplementary Material

Supplement 1

## Data Availability

The data used in this study are available by reasonable request under IRB approval and data use agreement with the respective data owners.
