# Supplementary material for: Novel Model of Childhood Appetitive Traits in Children with Obesity
Source: Res Sq. 2024 Nov 15:rs.3.rs-5318259. Preprint. [Version 1] doi: 10.21203/rs.3.rs-5318259/v1 (PMC11601821; doi:10.21203/rs.3.rs-5318259/v1)
Supplement: Supplement 1 [file NIHPPRS5318259v1-supplement-1.pdf]

Supplementary Files

This is a list of supplementary files associated with this preprint. Click to download.

- Table1.docx
- Table2.docx
- Supplemental.pdf
